# Human-prosthesis cooperation: combining adaptive prosthesis control with visual feedback guided gait

**DOI:** 10.1186/s12984-022-01118-z

**Published:** 2022-12-14

**Authors:** Bretta L. Fylstra, I-Chieh Lee, Minhan Li, Michael D. Lewek, He Huang

**Affiliations:** 1 Joint Department of Biomedical Engineering, North Carolina State University, Raleigh, NC 27695 USA; 2 Joint Department of Biomedical Engineering, University of North Carolina at Chapel Hill, Chapel Hill, NC 27599 USA; 3 Division of Physical Therapy, UNC Chapel Hill, Chapel Hill, NC 27599 USA

**Keywords:** Human-in-the-loop optimization, Transfemoral amputee, Powered prosthesis tuning, Visual feedback, Gait

## Abstract

**Background:**

Personalizing prosthesis control is often structured as human-in-the-loop optimization. However, gait performance is influenced by both human control and intelligent prosthesis control. Hence, we need to consider both human and prosthesis control, and their cooperation, to achieve desired gait patterns. In this study, we developed a novel paradigm that engages human gait control via user-fed visual feedback (FB) of stance time to cooperate with automatic prosthesis control tuning. Three initial questions were studied: (1) does user control of gait timing (via visual FB) help the prosthesis tuning algorithm to converge faster? (2) in turn, does the prosthesis control influence the user’s ability to reach and maintain the target stance time defined by the feedback? and (3) does the prosthesis control parameters tuned with extended stance time on prosthesis side allow the user to maintain this potentially beneficial behavior even after feedback is removed (short- and long-term retention)?

**Methods:**

A reinforcement learning algorithm was used to achieve prosthesis control to meet normative knee kinematics in walking. A visual FB system cued the user to control prosthesis-side stance time to facilitate the prosthesis tuning goal. Seven individuals without amputation (AB) and four individuals with transfemoral amputation (TFA) walked with a powered knee prosthesis on a treadmill. Participants completed prosthesis auto-tuning with three visual feedback conditions: no FB, self-selected stance time FB (SS FB), and increased stance time FB (Inc FB). The retention of FB effects was studied by comparing the gait performance across three different prosthesis controls, tuned with different visual FB.

**Results:**

(1) Human control of gait timing reduced the tuning duration in individuals without amputation, but not for individuals with TFA. (2) The change of prosthesis control did not influence users’ ability to reach and maintain the visual FB goal. (3) All participants increased their prosthesis-side stance time with the feedback and maintain it right after feedback was removed. However, in the post-test, the prosthesis control parameters tuned with visual FB only supported a few participants with longer stance time and better stance time symmetry.

**Conclusions:**

The study provides novel insights on human-prosthesis interaction when cooperating in walking, which may guide the future successful adoption of this paradigm in prosthesis control personalization or human-in-the-loop optimization to improve the prosthesis user’s gait performance.

## Background

Robotic lower limb prostheses have been an exciting research topic in the field of rehabilitation robotics [1–7]. Some devices have become commercially available, such as PowerKnee (Össur, Iceland) and Empower (Ottobock, Germany). These devices are usually designed to replicate the kinematics [2–7] and kinetics [1] of biologic joints in locomotion. Finite state machine control is the most used control approach, which operates the mechanics of prosthesis joints (often joint impedance, torque, or position) in different gait phases to assist locomotion. Theoretically, the desired joint mechanics (i.e., control parameters) are determined based on gait biomechanics observed in individuals without an amputation. However, in practice, when humans walk with a computer-controlled prosthesis (including passive devices, i.e., microprocessor prosthetic legs), the prosthesis control parameters must be tuned to adapt to different users due to inter-human variations in their physical conditions and gait pattern.

Personalization of robotic prosthesis control is often performed manually in labs or clinics [2–5, 8, 9], which is labor and time intensive and may lack accuracy. Further, the tuning procedure can become impractical with the increased number of control parameters needed to be tuned. Research groups have created and investigated their own automatic prosthesis tuning algorithms that adjust the control parameters (either joint impedance or torque profiles) of the prosthesis as the user is walking to achieve a pre-determined goal [10–13]. Focusing on transfemoral prostheses, our group designed model-free reinforcement learning (RL) algorithms to automatically tune 12 prosthesis control parameters to achieve normative knee motion in walking [12, 13], which has been a common tuning goal for research and commercial robotic prostheses [4, 5]. Through iterative prosthesis parameter tuning, these auto-tuning algorithms can successfully yield prosthesis kinematics, approximating the features of a desired knee profile (including joint peak values and timing) in gait within an error bound. However, it was noted that the peak values of knee kinematics converged more easily than the timing values. This is because the human user has input into the timing of the gait cycle (i.e., when to heel strike and when to toe-off) [13]. That is, even though the tuning goal for prosthesis control focuses on producing a prosthesis joint movement to assist a human user with walking, the prosthesis knee motion depends on both prosthesis control as well as on the human’s gait control. A similar observation was also reported in robotic ankle prostheses [14, 15], in which studies showed the importance of human-prosthesis coordination in terms of gait timing on prosthesis net ankle work and overall metabolic cost in human users. Addressing prosthesis control alone is insufficient to restore normative gait patterns; rather we need to consider both human gait and prosthesis control simultaneously [16].

Determining relevant human control parameters remains an important step in symbiotic human-device control. In short, what measures should be modified regarding the human’s gait pattern when walking with a lower limb prosthesis? In general, a shorter stance time on the prosthesis side is commonly observed in individuals with lower limb amputation in walking due to many reasons, such as motor weakness, change of inertia in the lower limb, lack of sensory feedback, maladaptation, etc. One way to modify the human’s gait pattern is to provide explicit instruction and feedback by, for example, physical therapists or biofeedback systems [17, 18]. To this end, our group has designed a visual FB system to guide individuals with amputation to extend their stance time on the prosthesis side while walking with a commercial powered knee prosthesis with fixed, manually-tuned control parameters [19, 20]. We showed that the visual FB system was effective in modifying gait timing in prosthesis users and accurately meeting the stance time goal. This visual FB tool enables us to investigate how prosthesis control tuning is affected as the human produces a more desired gait pattern (i.e., longer stance time on the prosthesis). Subsequently, we intend to determine how potentially different prosthesis control parameters can better assist the human user’s walking function.

Given the prior evidence that both human movement and prosthesis control influence prosthesis joint kinematics and overall gait [19, 21], we propose a new paradigm of human-prosthesis cooperation to achieve a desired gait pattern. The main goal of this study is to propose a new concept to the field of robotic lower limb prostheses—human-prosthesis cooperation. Pervious research has mainly focused on the “smart control” of robotic prostheses to meet a desired gait pattern; however, gait performance (e.g., gait symmetry), depends on both the prosthesis mechanics and human motor control of the un-amputated joints. Evidence has shown that the user does not necessarily coordinate their behavior with that of the robotic prosthesis in terms of timing and posture, which may explain the insufficient energy transfer from the robotic machine to the human user for improved walking energetic efficiency [8, 14, 22]. This evidence supports that as researchers, we cannot ignore human motor control while developing new robotic prostheses and the algorithms that control them. Therefore, we envision that human-prosthesis cooperation will enable a symbiotic relationship between both intelligent agents (human user and robotic prosthesis) to maximize mobility of the user. To our knowledge, research on human-prosthesis interaction and cooperation has been very limited. As a first attempt, this study, motivated by our previous studies, combined automatic tuning of prosthesis impedance control and visual FB of stance time together to enable human-prosthesis cooperation to achieve normative prosthesis knee kinematics and improved temporal symmetry in gait. We aimed to explore interactions between human and robotic prosthesis control, and their conjoined effects within the new paradigm of human-prosthesis cooperation. Specifically, we were interested in the following questions: (1) Does user control of gait timing (via visual FB) help the prosthesis tuning algorithm to converge faster? (2) In turn, does the tuning of prosthesis control influence the user’s ability to reach and maintain the visual FB goal in walking? (3) Does the prosthesis control parameters tuned while the user is in a more favorable gait pattern (i.e., extended stance time on the prosthesis side) allow the user to maintain this behavior even after feedback is removed? The first two questions explore the interaction of human gait control via visual FB and prosthesis adaptive control via reinforcement learning-based impedance tuning. The third question explores the potential benefit of human-prosthesis cooperation on overall gait patterns. The results from this study will contribute important knowledge in human-prosthesis interactions and may inform an effective way to address locomotion function in individuals with lower limb amputation wearing a robotic prosthesis.

## Methods

### Participants

Seven able-bodied, individuals without an amputation (AB) (age: 28 ± 8 yrs; mass: 72.2 ± 8.0 kg; and height: 1.76 ± 0.04 m) and four individuals with a unilateral transfemoral (or knee disarticulation) amputation (TFA) (age: 38 ± 17 yrs; mass: 82.5 ± 7.5 kg; and height: 1.76 ± 0.07 m) participated in this study (Table 1). Participants were recruited from the local community who were conveniently available to participate. Recruited participants had no known comorbidities that may affect their performance in this study. Participants provided written, informed consent to participate in this study approved by the University of North Carolina at Chapel Hill Institutional Review Board.

**Table 1:** Summary of participants

	AB01	AB02	AB03	AB04	AB05	AB06	AB07
Gender	Female	Male	Male	Male	Male	Male	Male
Height	1.78 m	1.75 m	1.79 m	1.75 m	1.72 m	1.70 m	1.83 m
Weight	83.9 kg	70.3 kg	65 kg	75.7 kg	79 kg	61 kg	70.3 kg
Age	23	21	45	29	24	31	24
Prosthesis side	Right	Left	Right	Left	Left	Left	Left
Previous experience with powered prosthesis?	No	No	Yes	Yes	No	No	No

	TF01	TF02	TF03	TF04
Gender	Male	Male	Male	Male
Height	1.68 m	1.73 m	1.83 m	1.80 m
Weight	83.9 kg	72.6 kg	82.9 kg	90.7 kg
Age	23	42	26	61
Prosthesis side	Left	Left	Right	Left
Type of amputation	Knee disarticulation	Transfemoral	Transfemoral	Transfemoral
Previous experience with powered prosthesis?	No	No	Yes	Yes
Time since amputation	18 years	3 years	9 years	49 years
Reason for amputation	Congenital	Trauma	Cancer	Cancer
Prescribed prosthesis	C-Leg 3 (Ottobock)	X3 (Ottobock)	X3 (Ottobock)	Genium (Ottobock)
Prosthesis suspension	Suction	Suction	Double-wall with pin to outer socket	Ischial containment suction
Self-reported Functional K-level	4	4	3	3

Seven individuals without amputation and four individuals with amputation participated in this study. Seven participants had no prior experience with our powered prostheses and underwent training with the device for 1–5 days

### Prosthesis training and acclimation

**Fig. 1:**
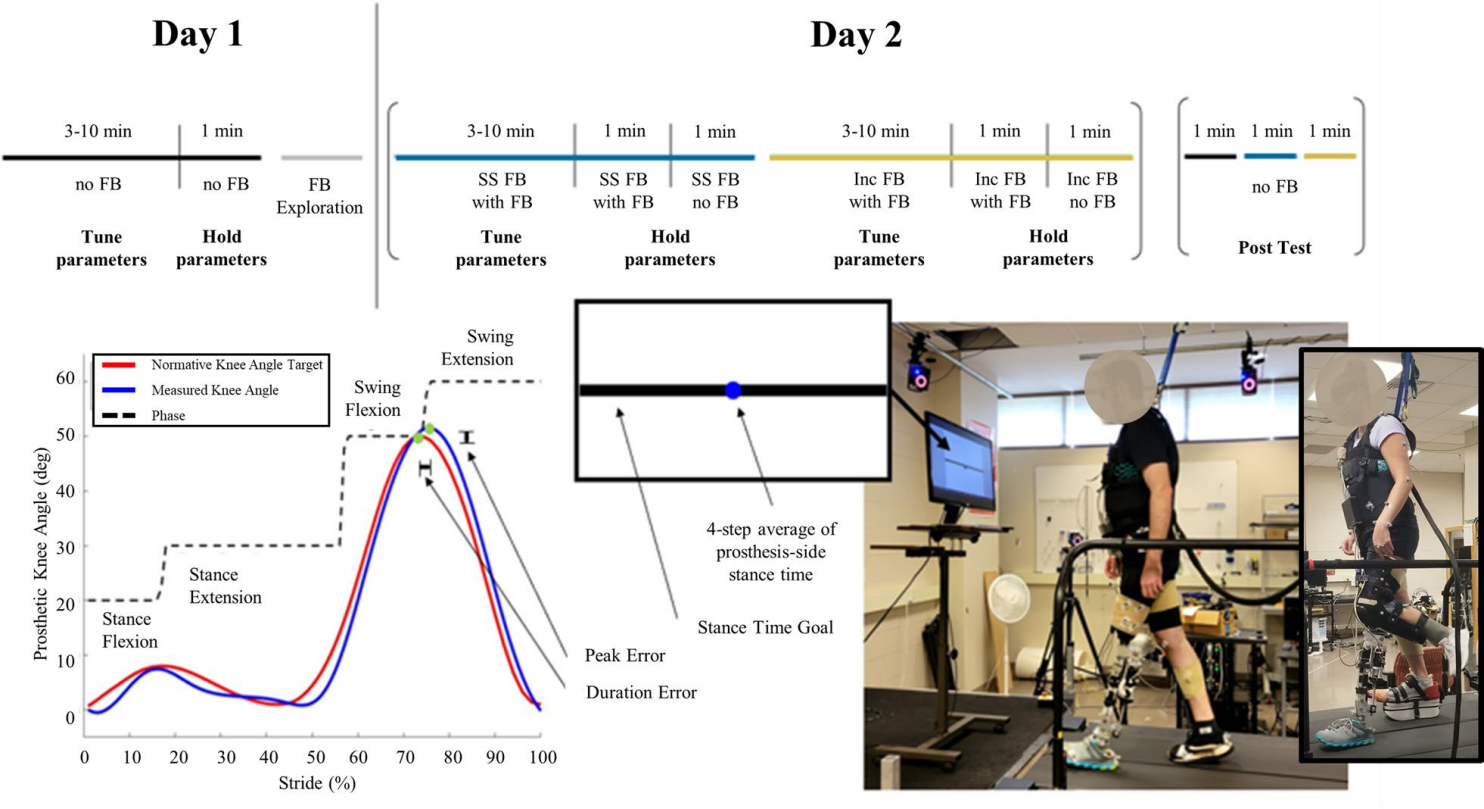
(Top) Explanation of methods. Listed all condition on day 1 and day 2. (Bottom left) Explanation of prosthesis tuning. The red line shows the target knee profile and the blue line shows an example knee profile during tuning. Peak and duration errors were calculated for each phase as shown. (Bottom right) Explanation of the visual feedback. The participant was given feedback of the stance time of their prosthetic limb averaged over the previous four steps

All participants were trained to walk with the powered knee prosthesis on a treadmill. The purpose of training was to ensure that participants were able to adapt to the powered knee prosthesis, be confident to walk with the device, and produce a consistent gait pattern. Participants without an amputation donned an L-shaped, bent-knee adapter (Fig. 1) to connect to the powered prosthesis and were trained for at least five visits. The prosthesis alignment was done following the L.A.S.A.R. protocol [23], and a shoe lift was used to ensure the hips were level. For the participants with TFA, a certified prosthetist assisted with the alignment of the prosthesis and then underwent 1–2 days of training. During training, the desired knee impedance parameters were hand tuned by an experienced experimenter to allow comfortable and safe walking. After training, all participants were able to achieve steady-state walking at 0.6 m/s, were able to adapt to the powered prosthesis, and generate a stable and consistent gait pattern. Two of the four participants with TFA had previous experience walking with the prosthesis. They were given approximately 20 min to re-acclimate to the device and fine-tune the parameters as needed.

### Experimental protocol

After training, participants returned to the lab twice to complete the conditions detailed in Fig. 1. A reinforcement learning-based framework developed by our team [12] was applied to auto tune the impedance control parameters (see “Prosthesis tuning” section for details). Participants were instructed to walk as consistently as possible on a treadmill while the control parameters updated every four gait cycles until the knee angle profile was within the bound. After tuning converged, a 2-min walking trial with the final tuned parameters was conducted. In this study, three sets of control parameters: auto-tuning without visual feedback (No FB), auto-tuning with self-selected stance time visual feedback (SS FB) and auto-tuning with increased stance time visual feedback (Inc FB) were tuned.

The visual FB protocol was similar to Brandt et. al [19, 20] (see “Visual feedback” section for details). The self-selected and increased stance time were determined for each participant and then displayed on a monitor as the visual feedback (visual FB) goal. During the SS FB and Inc FB conditions, while tuning the control parameters, participants were asked to control their stance time as shown on the monitor to match with the determined timing goal. After tuning converged, participants walked with the tuned parameters for a 2-min trial where the feedback was displayed for the first minute and then removed for the second minute. A post-test was performed after 10 min of rest to investigate if the stance time could be maintained after the visual FB was removed. Participants completed three, 1-min no feedback trials, walking with the fine-tuned parameters determined in the No FB, SS FB, and Inc FB conditions.

On the first day, the No FB condition was completed, and the self-selected and increased stance times were determined. During the second day, the remaining two conditions (SS FB and Inc FB) and the post-test conditions were conducted. The order of the SS FB and Inc FB were randomized with roughly half of the participants receiving each condition first. The order of No FB, SS FB, and Inc FB in the post-test section was randomized.

Gait kinematics and kinetics were measured. Kinematics were captured with 43 light-reflective markers placed on the bony landmarks of the acromia, iliac crests, greater trochanters, anterior and posterior superior iliac spine, medial and lateral femoral epicondyles, medial and lateral malleoli, first and fifth metatarsals, and calcanea to define the torso, pelvis, thighs, shanks, and feet segments, respectively with the addition of tracking markers on each segment. A 12-camera motion capture system (VICON, Oxford, UK) sampled at 100 Hz, captured marker data and bilateral ground reaction forces (GRFs) were synchronously recorded via a Bertec split-belt treadmill (Bertec Corp, Columbus, OH, USA) sampled at 1000 Hz.

### Prosthesis tuning

For the auto-tuning trials, the prosthesis control parameters were adjusted every four strides (counted as one iteration) to achieve a normative knee angle profile [24] (Fig. 1). We characterized the profile with four discrete feature points, with each point representing a local extremum in the corresponding gait phase along the profile. A total of 12 impedance control parameters (stiffness, equilibrium position, and damping for each of the four gait phases—stance flexion, stance extension, swing flexion, and swing extension) were adjusted to match the normative knee angle profile. The adjustment was realized by an existing reinforcement learning algorithm as detailed in Li et al. [12], and it was considered a success when the tuning convergence criteria were met, where the peak and duration errors between the measured and normative knee angle profiles (as defined in Fig. 1) were within 2° and 3% (i.e., target range), respectively, for 8 out of 10 consecutive iterations.

During auto-tuning trials, participants walked in bouts of approximately two minutes consisting of 10 iterations (40 strides) to prevent fatigue. In the case of the Inc FB condition, after each bout of 10 iterations, the duration error was calculated and if 8/10 of the iterations were not in the target range the stance phase of the normative knee angle profile was increased by 2% to accommodate changes in the knee profile curve due to the increased stance time FB goal. This allows the prosthesis tuning to adapt to the user’s changing behavior.

### Visual feedback

Based on the protocol in Brandt et al. [19], prosthesis-side stance time was used as the visual FB. The visual FB was created via custom code using the Vicon DataStream SDK (VICON, Oxford, UK) and MATLAB (The MathWorks, Inc, Natick, Massachusetts, USA) and displayed on a computer monitor, approximately one meter in front of the participant on the treadmill. Prosthesis-side stance time was calculated from the real-time GRF signals using a 20N threshold. The feedback displayed to the user was averaged over four strides to reduce any large stride-to-stride corrections.

To determine the visual feedback goals, the participant’s stance time was first calculated from the auto-tuning No FB condition and presented as their SS FB goal. Participants could then adjust the feedback goal, making the preferred stance time longer or shorter, during the exploration session on day 1 until they felt comfortable—this final stance time was their self-selected stance time. The visual FB level was then increased by increments of 0.05 s until participants reported an increased rate of perceived exertion and they could reliably maintain the target stance time. Most participant’s increased stance time goal was 0.10 to 0.15 s longer than their self-selected stance time. Both targets were centered on the screen and the display range remained at ± 0.3 s from the target line to maintain participant’s perceived accuracy.

### Data processing

Commercial data analysis software (Visual 3D, C-Motion, Inc., Germantown, MD, USA) was used to process the data. Marker data were low-pass filtered with a 4th order Butterworth filter with a cutoff frequency of 6 Hz and GRF signals were smoothed by a 4th order Butterworth filter with a cutoff frequency of 25 Hz. Gait events (heel contact and toe-off) were determined with a threshold of 20N. To determine gait symmetry, we used a standard symmetry index where ${x}_{i}$ and ${x}_{p}$ are the stance time of the intact and prosthesis sides, respectively.

$$SI= \frac{{x}_{i}-{x}_{p}}{\left({x}_{i}+{x}_{p}\right)/2}*100\%$$

Target-hitting error was defined as the absolute value of the time between the goal stance time and the user’s prosthesis-side stance time and the target-hitting variability was the standard deviation of their error. Extrapolated center of mass (xCOM) was calculated using the equation from Hof et al. [25], which was developed for individuals with transfemoral amputation where z, g, and h refer to the lateral position of the COM (m), gravitational acceleration, and 1.34 times leg length (m). Margin of stability (MOS) was estimated using the heel marker of each foot as the base of support (BOS).

$$xCOM\left(t\right)=z(t)+ \frac{1}{\sqrt{g/h}}\cdot \frac{dz}{dt}$$

$$MOS=xCOM-BOS$$

### Statistical tests

To answer the first question, if tuning with visual FB facilitates faster tuning, we performed a two-way cross tabulation Chi-square test for independence to examine if there is a relationship between populations (TFA and AB) and feedback level (no FB and SS FB). If the Chi-square test reaches significance, the adjusted residual will be used for the post-hoc comparison (the z critical values of ± 1.96 were set as the significance level). To answer the second question, if tuning affects feedback performance, pooled t-tests were used to compare error and error variability during tuning and during steady-state for both the SS FB and Inc FB conditions. Finally, to answer our third question, if participants can retain their increased stance time and better stance time symmetry after feedback was removed, two-way mixed model ANOVAs were used including participants as random effects and feedback, population, and their crossed effects as fixed effects. We compared across the population (TFA vs. AB) and across the Inc FB conditions [with FB, immediately after FB, and long after FB (post-test)] as well as across the post test conditions (no FB—post, SS FB—post, and Inc FB—post). For the fixed effects that reached the significant level, Tukey’s honestly significant difference test was used for the post hoc comparisons. A follow-up within participant analysis was conducted using the Friedman non-parametric test. A non-parametric test was chosen because the samples within participant were non-independent samples (i.e., consecutive walking steps). The Shapiro–Wilk test was used to test the normality before running the ANOVAs. The significance level was set at α = 0.05, and all analyses were performed using JMP (JMP, SAS Institute, Cary, NC, USA).

## Results

### Time to tune the prosthesis

**Fig. 2:**
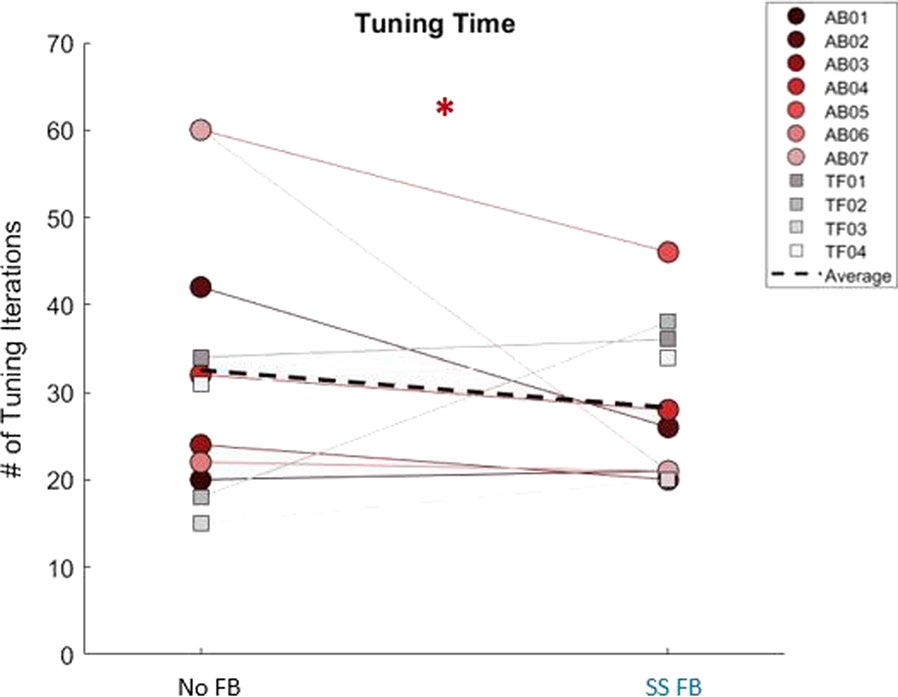
Number of tuning iterations with and without feedback. Many participants had similar tuning times with and without feedback; however, some participants had noticeable decreases in tuning time when tuning with preferred stance time visual feedback. One amputee participant (TF02) had a noticeably longer tuning time with feedback

We defined the tuning performance as the number of tuning iterations during tuning without FB and with self-selected stance time FB (Fig. 2). There was a significant effect both for population and feedback (${\rm X}_{(1)}^{2}= 14.134$, *p* < 0.0001). The comparison between the feedback level in the population without an amputation showed that the no FB condition required significantly more tuning iterations than the SS FB condition (adj. residual: no FB 22.9 and SS FB − 22.9). In addition, in the population with an amputation, a significantly longer tuning time was required with feedback compared to without feedback (adj. residual: no FB − 22.9 and SS FB 22.9).

**Fig. 3:**
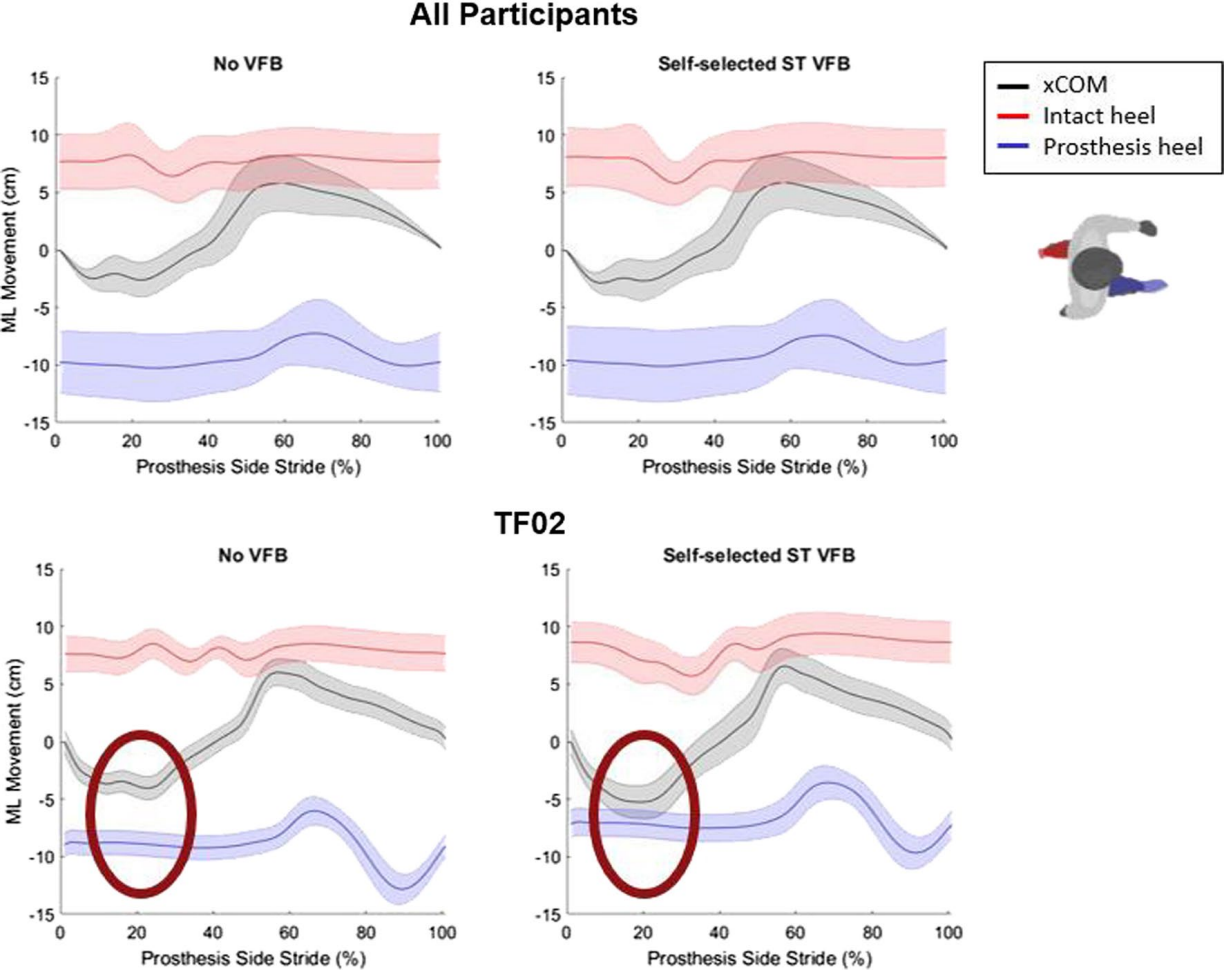
(Top) Extrapolated center of mass (xCOM) relative to the prosthesis and intact heel marker positions averaged across all 11 participants. (Bottom) xCOM relative to the prosthesis and intact heel marker positions for TF02. On average, there is no discernable difference in balance between auto-tuned parameters without feedback and auto-tuned parameters with self-selected stance time feedback. However, for participant TF02 who had a noticeable increase in tuning time with feedback, the xCOM had greater medial–lateral movement and was closer to the bounds of the base of support

When investigating an explanation for why tuning was longer with FB for TF02 (who showed the largest increase across all participants), we investigated balance measures. Figure 3 shows the medial–lateral position of the extrapolated center of mass (xCOM) relative to the base of support (BOS) as defined by the prosthesis and intact limb heel markers over the prosthesis stride. On average, there was no noticeable change in the xCOM movement in relation to the base of support. However, TF02 experienced a 23% decrease in the average margin of stability (average difference between the xCOM and prosthesis-side BOS per stride) when the parameters were tuned with FB.

### Visual feedback target accuracy

**Fig. 4:**
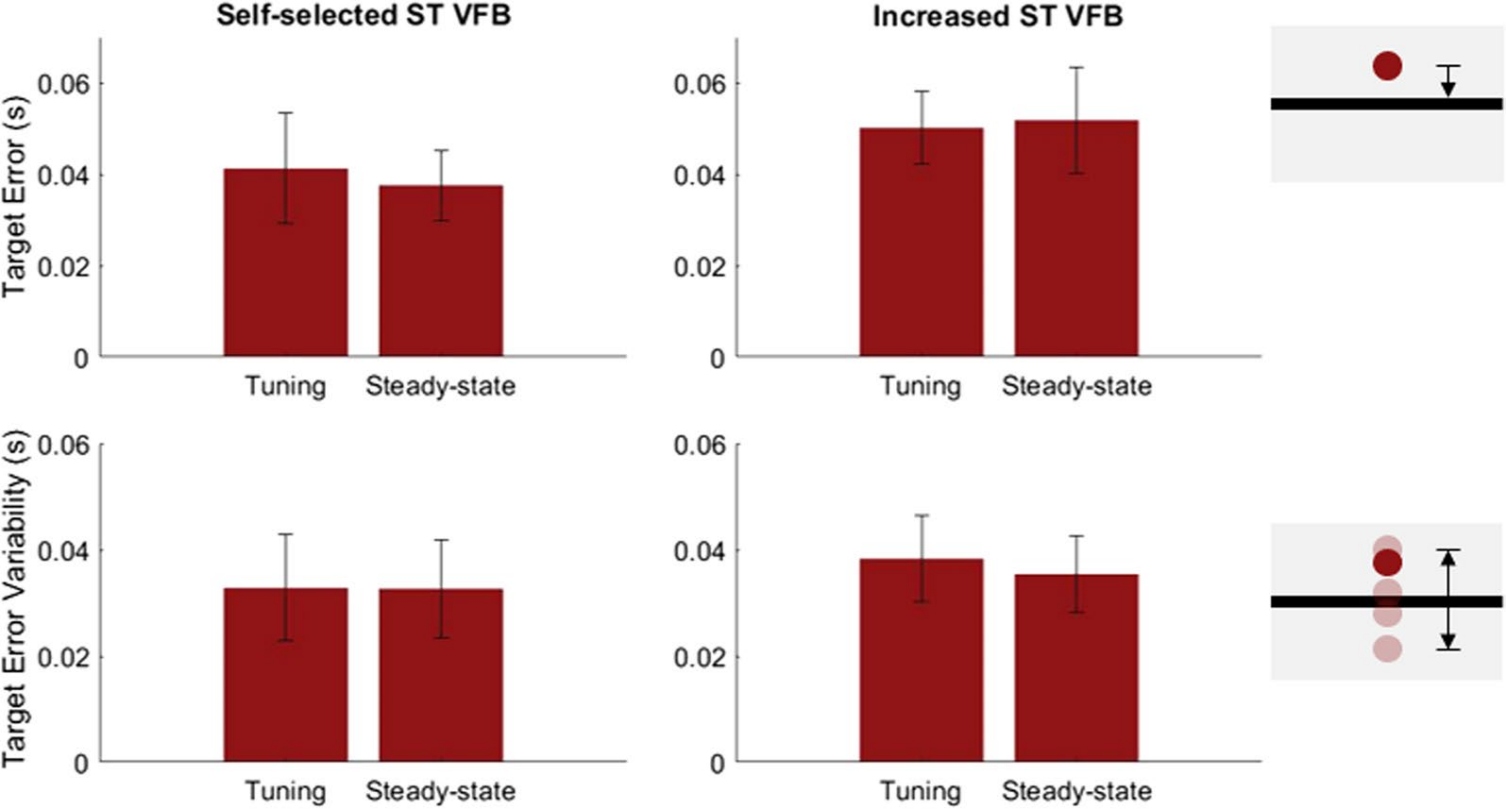
(Top) Absolute value of the target error during tuning with self-selected stance time visual feedback and increased stance time visual feedback and during steady-state. (Bottom) standard deviation of the target error. While tuning the prosthesis parameters, the users’ performance to hit the target and variability in hitting the target was not different from their steady-state performance. Pictures to the right depict the error and error variability. The horizontal line was the goal stance time and the red dot represents the averaged prosthesis side stance time. The arrows show the target error (top 2 plots) and error variability (bottom 2 plots)

We defined feedback performance as the absolute value of the error between the user’s stance time and the goal, as well as the variability around the goal (standard deviation of the absolute error) as seen in Fig. 4. For the two trials with feedback (self-selected and increased stance time FB), there was no significant difference in the error (p = 0.1149 for SS FB and p = 0.4471 for Inc FB) or variability during tuning and during steady-state (p = 0.9660 for SS FB and p = 0.1527 for Inc FB).

### Stance time and stance time symmetry: retention effect

**Fig. 5:**
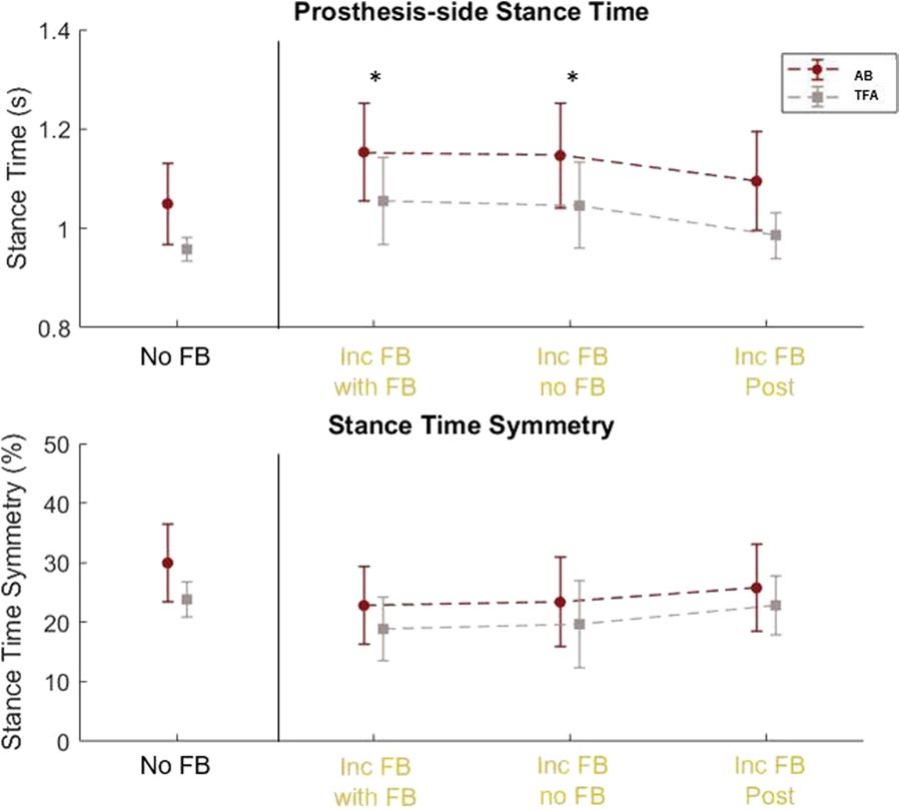
Prosthesis-side stance time and stance time symmetry for all participants during no visual feedback (No FB), increased stance time visual feedback during feedback (Inc FB with FB), increased stance time visual feedback after feedback was removed (Inc FB no FB), and increased stance time visual feedback in the post-test (Inc FB Post). All participants were able to increase their prosthesis-side stance time with the feedback and maintain it after feedback was removed. However, this longer stance time was not retained in the post-test. *Indicates significantly different from Inc FB Post

Figure 5 shows stance time and stance time symmetry for both populations across the baseline condition, Inc FB with FB, Inc FB no FB, and Inc FB post. All participants significantly increased their stance time (p = 0.0021; 1.05 s → 1.15 s for AB participants and 0.96 → 1.06 s for TFA participants) and decreased their stance time asymmetry (30% → 23% for AB participants and 24% → 19% for TFA participants). In a post-hoc analysis, the stance time was significantly longer in the Inc FB with FB and Inc FB no FB conditions compared to the post-test with Inc FB (p = 0.0032 for Inc FB with FB and p = 0.0089 for Inc FB no FB). Further, no significant difference was found between the two populations (participants with and without an amputation).

### Stance time and stance time symmetry in the post test

**Fig. 6:**
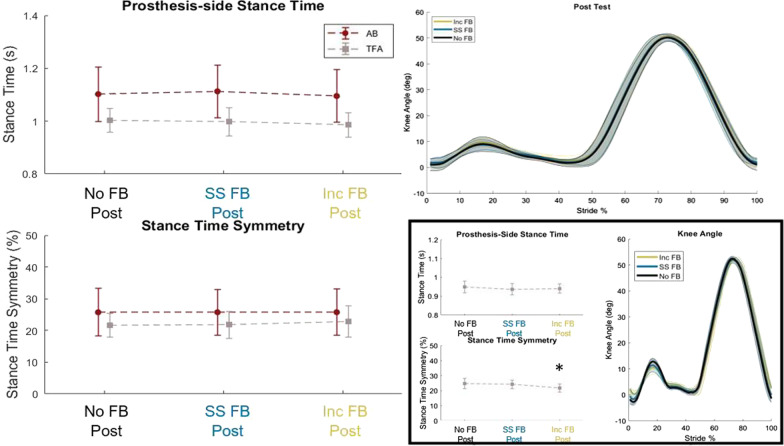
(Left) Prosthesis-side stance time and stance time symmetry averaged across all participants for the three post-test conditions. The post-test compared the three different control parameter sets that were determined after tuning without feedback (No FB Post), with self-selected stance time feedback (SS FB Post), and with increased stance time feedback (Inc FB Post). There was no significant difference across conditions. (Right Top) Knee angle averaged across all participants for the three post-test conditions. (Right Bottom) One representative TFA participant’s stance time, stance time symmetry, and knee angle profile in the three post-test conditions. Although there was no significant difference across conditions, the result of the individual analysis showed that roughly half the participants experienced a significant difference in the stance time or stance time symmetry. We observed that these participants had variations in their knee angle profiles for the different tuning conditions as representatively shown in the bottom right

Across all participants, there were no significant differences in stance time and stance time symmetry between the three post-test conditions (no FB, SS FB, and Inc FB); however, the participants (modeled as a random effect in the two-way mixed model ANOVA) reached significant differences (p = 0.0373 for stance time and p = 0.0370 for stance time symmetry). To further investigate this significant effect on the individual participants, we ran a follow-up analysis comparing the three post-test conditions within participant. Three participants (AB04, AB05, and TF01) had significantly longer stance time in SS FB or Inc FB than walking with the no FB parameters (ps < 0.0443) and TF03 had a significantly better stance time symmetry (p = 0.0019). To further investigate the individual differences, the knee angle profiles in the three post-test conditions were examined (Fig. 6). From visual inspection, we can see that for these previously named participants, there are subtle differences in the stance flexion peak between the three tuned parameter sets. A larger stance flexion peak is associated with a longer stance time. This may explain the individual differences in stance time and stance time symmetry.

## Discussion

In this study, we proposed a novel paradigm for human-prosthesis cooperation to produce normative prosthesis knee motion in walking. A visual feedback system provided the goal of desired stance time for human users to modify their gait timing, while the prosthetic controller auto-tuned the impedance parameters to meet the desired joint magnitude and timing at different gait phases. Under this novel paradigm, we investigated (1) how the paradigm influences prosthetic tuning and human timing control individually, and (2) when the stance time is controlled in a favorable way (with longer stance time on the prosthesis side), does the tuned controlled parameters better support gait? The first question is crucial to understanding human-prosthesis interaction and the feasibility of this paradigm for human-prosthesis cooperation. The knowledge of the second question aims to know whether our human-prosthesis cooperation paradigm can benefit prosthesis control personalization to better support the user’s gait pattern.

Our results suggested that prosthesis control tuning did not influence the human’s ability to achieve their gait timing control, cued by a visual FB system. All participants were able to control the stance time precisely and accurately based on the provided visual FB target, regardless of whether the prosthesis tuning (adaptation) was activated. Although prosthesis tuning can lead to changes in prosthesis joint mechanics, the human user maintained control of the targeted stance time. This implies that humans, compared to computerized prosthesis control, dominate the timing control in gait. In short, the human’s intention and action can override the influence in mechanical changes of the prosthesis knee on gait timing and timing is a vital part of prosthesis control. Both the user and the prosthesis need to be aligned temporally to allow the prosthesis to accurately and harmoniously deliver the correct power and kinematics to the user.

Similarly, human control of gait timing did not negatively impact the ability of RL-based prosthesis tuning to converge, but it did not accelerate the prosthesis tuning process to meet the normative gait kinematics either. We initially expected that if humans were able to successfully control gait timing through visual FB, fewer tuning iterations would be observed before the convergence of the knee kinematic features (magnitude and timing) to the desired target value. However, only participants without an amputation met this expectation. When the participants with an amputation were asked to control stance time, slightly more tuning iterations were needed for 3 out of 4 participants (less than 5 iterations), and about 20 more for TF02 (see Fig. 2). We suspect that this was caused by a change of gait dynamics and pattern in individuals with amputation when they walked with visual FB. Clear evidence was observed in TF02, who showed a reduction in the margin of stability with visual FB, compared to that without visual FB. Such a change in gait pattern with visual FB challenged the prosthesis tuning policy (learned when visual FB was off) to quickly accomplish the tuning task. Although we previously showed that our RL-learned policy for prosthesis control is robust and can be generalized to other gait patterns [12], additional iterations and time to converge was needed when the dynamics drastically changed. Therefore, we postulate that adding a human control goal (i.e., gait timing control in this study) that aligns with the machine tuning goal may improve prosthesis tuning speed if the added human control goal does not significantly change the overall gait dynamics and pattern of the prosthesis user. This can be our future work to validate.

Why did visual FB cause the gait pattern change in TF02? Even though the targeted stance time matches their preferred stance time, TF02 probably experienced difficulty in the task to maintain the gait timing from step to step and, therefore, developed a significantly different motor pattern to compensate. Another potential explanation was that visual FB occupied additional cognitive resources. It has been known that individuals with amputation, compared to individuals without an amputation, demand higher cognitive resources during regular walking to pay attention to the environment and their own body control [26, 27]. Visual FB introduced a secondary task goal for the individuals with amputation to achieve [28], which may lead to a less stable gait pattern. Therefore, to prevent gait dynamic/pattern change, we should explore other biofeedback modalities (e.g., vibrotactile, sounds, electrostimulation, etc.) and timing (e.g., continuous vs. intermittent feedback) to identify the biofeedback design that is more intuitive to individuals with an amputation. In addition, physical therapists may assist the prosthesis user with practicing walking with visual FB to ensure that participants do not develop compensations and drastically change their gait pattern to ensure time efficient prosthesis tuning under our proposed human-prosthesis cooperation paradigm.

The retention effects in the same experimental session were only observed right after the visual FB was removed. Similar to Brandt et al. [19], participants were able to increase their prosthesis-side stance time and improve their stance time symmetry index with the visual FB that cued extended stance duration. We further examined the retention of visual FB effects immediately and 10 min after the visual FB session in this study, respectively. Even though the participants were not explicitly instructed to maintain their increased stance time right after the visual FB session, they were able to maintain the timing symmetry. However, after a 10-min break, the participants reverted to approximately their baseline stance time on the prosthesis side and stance time symmetry in the post-test trials (see Fig. 5). This result indicated that the effect of visual FB on extended stance time control was gradually removed in a short period. This may be caused by many factors, such as motor weakness and increased efforts in postural control, which were not preferred by the prosthesis users [20]. In the future, visual FB training may be combined with other therapeutic goals, such as strengthening of residual joints and balance training, in order to evaluate the potential benefit of visual FB in human gait training.

Combining auto-tuning of the prosthesis control with extended prosthesis-side stance time (i.e., improved temporal symmetry) visual FB did not yield prosthesis control to support better gait symmetry, compared to tuned control without visual FB and tuned control with self-selected stance time visual FB. These results provide additional evidence that (1) humans have the tendency to maintain their intrinsically preferred gait temporal pattern, and (2) the effects of prosthesis control on gait timing can be easily overridden by the human’s gait pattern. That is to say, human gait control dominates the gait timing. This timing is vital to ensure that the prosthesis power and device trajectory aligns with the user to enable smooth and efficient gait. Interestingly, when we examined individual participants’ data, we found that 4 out of 11 participants (2 AB and 2 TFA) did observe a significantly longer stance time and/or better stance time symmetry while walking with the Inc FB or SS FB control parameters and was associated with the stance flexion peak of the knee profile (see Fig. 6). Perhaps prosthesis tuning to achieve normative knee kinematics cannot improve humans’ gait symmetry; rather a different knee kinematic profile is needed. In addition, the knee profile may need to be customized to each user to account for different limb geometry such as knee disarticulation and residual limb length for individuals with transfemoral amputation.

Our observation in this study may explain why previous studies on human-in-the-loop optimization (HILO) of prosthesis control did not always show significant gait performance improvement for individuals with an amputation [8, 11]. For example, one study unsuccessfully used HILO to attempt to optimize prosthesis ankle control torque to minimize the metabolic cost (as the cost function) [8]. Our study may offer insights to explain the phenomena. We showed that the humans’ goal and associated gait pattern may have a greater contribution to some gait performance metrics than the prosthesis control. That means the human’s goal for the generated gait pattern may dominate the performance measures (e.g., metabolic cost) of the human-prosthesis system and is capable of overriding the effects of prosthesis control on those measures. Since the existing studies on HILO of prosthesis control do not specify the human goal regarding how to interact with the powered prosthesis in walking, it could be any goal that the individual intends/prefers rather than energy reduction, as is commonly assumed. For example, if the prosthesis user’s goal (say maintaining balance) during walking increases the chosen HILO cost function (e.g., metabolic cost), the cost function may not converge. We can speculate that consideration of the human’s walking goal (independent of metabolic cost) may improve HILO prosthesis control. The open question is what gait control goals should be assigned to humans and prostheses controllers to achieve improved gait performance in prosthesis users. One potential approach is to use inverse reinforcement learning (IRL) considering the cooperative and interactive nature of the human–robot system to construct a control objective to account for goals for both humans and robot [29]. Another approach is to let users adjust prosthesis control so that the prosthesis action is aligned with the intrinsic goal of human users [30]. Continued research efforts and innovative ideas are needed to determine appropriate collective human-prosthesis goals to achieve an optimized gait of individuals with amputation when walking with intelligent robotic prostheses.

In addition, our proposed paradigm can be extended to other existing wearable devices and controllers [10, 31]. Although our specific formulation of human-prosthesis cooperation may not be directly applicable to other devices or control, the proposed human-prosthesis cooperation paradigm can be generalized for exploring various human biofeedback and prosthesis tuning goals. For example, in the case of human-in-the-loop optimization to reduce the metabolic cost of walking, providing biofeedback to the user of metabolic cost may affect the algorithm’s convergence speed because both the human and prosthesis are working towards a common goal of reduced metabolic cost. Similarly, providing biofeedback of medial–lateral balance or prosthesis push-off work could improve algorithm convergence time because large sways in balance and diminished work at the ankle during the push-off phase could contribute to increases in metabolic cost. Passing this control of balance or work to the user may improve overall gait performance and algorithm speed. As another example, an echo-control scheme that aims to match the contralateral limb may benefit from biofeedback to the user of stance time or step length symmetry. In this case, both the user and device are focused on matching the contralateral limb which again, may improve performance. Our results presented here demonstrate one initial idea of combining stance time and knee kinematics to improve algorithm convergence and although we observed inconsistent results between our two populations, this framework of human-prosthesis cooperation offers researchers a platform to further explore and modify to fit their own potential algorithm pitfalls.

Finally, although we were able to recruit 11 participants, seven of our participants were individuals without an amputation wearing a prosthesis adapter. As shown in previous studies, results from individuals without amputation may not completely translate to individuals with amputation [8] possibly due to the individual’s limited experience with the prosthesis. Therefore, systematically testing more individuals with amputation is necessary. We included results from both populations to further emphasize the similarities and differences between these populations. It is also noted that all participants first completed the No FB condition on day one and then completed the SS FB and Inc FB on day 2. Participants completed the No FB condition first so that the FB would not influence their typical walking pattern. All participants received prior training and had to reach a stable gait pattern to participate in the study, so it is unlikely any additional learning would occur between the 2 days. In addition, although, as the first attempt in studying human-prosthesis cooperation, our paradigm opens the door for more researchers to explore human-prosthesis interactions, here we focused on the feedback of prosthesis-side stance time with respect to a consistent target and tuning prosthesis control to a normalized knee angle profile. With such a preliminary design, despite the interesting findings discussed above, we did not find the clear benefit of providing a visual FB goal on prosthesis tuning and overall gait performance. Additional research on how to leverage this cooperation paradigm to facilitate prosthesis control personalization or overall human gait performance is necessary.

## Conclusion

This study presents a novel paradigm for tuning and personalizing robotic prosthesis control. This paradigm engaged both intelligent prosthesis control to tune the machine mechanics and human control of gait via biofeedback to cooperate and facilitate the prosthesis tuning goal in walking. This paradigm enabled us to examine human-prosthesis interaction. Human or prosthesis adaptation did not significantly influence each other, showing the feasibility of this paradigm to enable human-prosthesis cooperation and co-adaptation. However, as the first attempt in designing and implementing this paradigm, we did not find clear benefit of human control in the prosthesis tuning loop. Additional research is still needed to understand the mechanism of human-prosthesis interaction in walking and identify and implement appropriate human-prosthesis collective goals to benefit prosthesis personalization procedure and overall gait performance for individuals with amputation.

## Data Availability

All of the data and material is owned by the authors and/or no permissions are required.
